# Epileptic seizure? Certainly uncertain

**DOI:** 10.1007/s10633-021-09839-7

**Published:** 2021-04-30

**Authors:** Sven P. Heinrich, Hansjürgen Agostini

**Affiliations:** 1 Eye Center, Medical Center, University of Freiburg, Killianstr. 5, 79106 Freiburg, Germany; 2 Faculty of Medicine, University of Freiburg, Freiburg, Germany

Dear Editor,

We appreciate the cautionary remarks by Hamilton and Zuberi [1] in response to our recent case report [2]. They express concerns that the presented case had been labelled as “epileptic” in the absence of sufficient evidence.

Indeed, the exact nature of the event is unclear. The circumstances prevented a direct observation of the development of symptoms, and we had no influence on the subsequent examinations that were performed routinely in the neuropediatric unit. As per the patient files, no EEG was performed.

Given this uncertainty, we carefully avoided using “epilepsy” or “epileptic” when describing the event itself. Of course, we could not avoid using these terms when providing a larger context, referring to the literature, or addressing the patient history. We regret that this may have caused the impression that we were certain about the classification of the event.

We are grateful to Hamilton and Zuberi for pointing us to the CEVnet archive.
